# Quasi-static mechanical evaluation of canine cementless total hip replacement broaches: effect of tooth design on broach and stem insertion

**DOI:** 10.1186/s12917-024-04075-y

**Published:** 2024-05-24

**Authors:** Zachary T. Lawson, Danielle L. Hollenbeck, Catrina J. Silveira, Michael R. Moreno, Andrew B. Robbins, W. Brian Saunders

**Affiliations:** 1 College of Engineering, Texas A&M University, College Station, TX USA; 2 Department of Small Animal Clinical Sciences, College of Veterinary Medicine and Biomedical Sciences, 4474 TAMU, College Station, TX 77843-4474 USA

**Keywords:** Total hip replacement, THR, Canine, Cementless, Press-fit, Broach, Broach tooth design, Quasi-static, Biomechanics

## Abstract

**Background:**

Biomedtrix BFX^®^ cementless total hip replacement (THR) requires the use of femoral broaches to prepare a press-fit envelope within the femur for subsequent stem insertion. Current broaches contain teeth that crush and remove cancellous bone; however, they are not particularly well-suited for broaching sclerotic (corticalized) cancellous bone. In this study, three tooth designs [Control, TG1 (additional V-grooves), TG2 (diamond tooth pattern)] were evaluated with a quasi-static testing protocol and polyurethane test blocks simulating normal and sclerotic bone. To mimic clinical broaching, a series of five sequential broach insertions were used to determine cumulative broaching energy (J) and peak loads during broach insertion. To determine the effect of broach tooth design on THR stem insertion, a BFX^®^ stem was inserted into prepared test blocks and insertion and subsidence energy and peak loads were determined.

**Results:**

Broach tooth design led to significant differences in broaching energy and peak broaching loads in test blocks of both densities. In low density test blocks, TG1 required the lowest cumulative broaching energy (10.76 ±0.29 J), followed by Control (12.18 ±1.20 J) and TG2 (16.66 ±0.78 J) broaches. In high density test blocks, TG1 required the lowest cumulative broaching energy (32.60 ±2.54 J) as compared to Control (33.25 ±2.16 J) and TG2 (59.97 ±3.07 J).  During stem insertion and subsidence testing, stem insertion energy for high density test blocks prepared with Control broaches was 14.53 ± 0.81 J, which was significantly lower than blocks prepared with TG1 (22.53 ± 1.04 J) or TG2 (19.38 ± 3.00 J) broaches. For stem subsidence testing in high density blocks, TG1 prepared blocks required the highest amount of energy to undergo subsidence (14.49 ± 0.49 J), which was significantly greater than test blocks prepared with Control (11.09 ±0.09 J) or TG2 (12.57 ± 0.81 J) broaches.

**Conclusions:**

The additional V-grooves in TG1 broaches demonstrated improved broaching performance while also generating press-fit envelopes that were more resistant to stem insertion and subsidence. TG1 broaches may prove useful in the clinical setting; however additional studies that more closely simulate clinical broach impaction are necessary prior to making widespread changes to THR broaches.

**Supplementary Information:**

The online version contains supplementary material available at 10.1186/s12917-024-04075-y.

## Background

Total hip replacement (THR) is a highly-effective treatment for pathologies of the canine hip such as hip dysplasia, femoral head or neck fracture, acetabular fracture, avascular necrosis of the femoral head, and traumatic hip luxation [1–4]. Advances in canine THR technology have followed a similar path as with human THR. While cemented THR implants were the standard of care in small animal orthopedics for several decades [1, 5], cementless implants were subsequently developed and are now commonly used [6–11]. Press-fit cementless implants such as the Biomedtrix BFX® system rely on a precision preparation and implantation technique leading to direct contact between the implant and adjacent bone [10, 12]. After impaction of the implant into the preparation bed, initial stability is provided by friction between the surface of the implant and the surrounding bone. Long-term stability develops through processes such as bone ongrowth and ingrowth [13, 14].

While technique and instrumentation vary somewhat between cementless implant systems, femoral canal preparation for press-fit cementless stems is accomplished with an incremental series of instruments referred to as femoral broaches. Femoral broaching is guided pre-operatively by radiographic templating and intra-operatively as the surgeon encounters an increase in the difficulty of broach insertion [12]. Broaches contain teeth that both crush and remove cancellous bone and are designed such that their external geometry approximates the dimensions of size-matched, press-fit, cementless stems [15]. Proper femoral broaching involves precise, consistent broach insertion along a pre-determined broaching pathway using an impaction mallet [16]. When performed properly, broaching generates a press-fit envelope of bone which circumferentially contacts the surface of the stem and limits stem subsidence. Subsidence is defined as the distal movement of a press-fit cementless stem within the femur in the early post-operative period. Improper broaching technique, insufficient percentage of canal fill, the presence of poor cancellous bone quality, or a low canal flare index (CFI) can independently or collectively contribute to insufficient press-fit and increase the risk of stem subsidence in the early postoperative period. The untoward consequences of stem subsidence include fibrous tissue ingrowth, prosthesis luxation, and femur fracture [17–19].

Intra-operative femoral fissure and post-operative femoral fracture are well-known complications associated with cementless THR. Femur fractures are caused by broach malalignment, impaction of an oversized broach, application of excessive force during broaching, depletion of cancellous bone during femoral neck osteotomy, or the presence of certain femoral morphologies such as low CFI or thin femoral cortices [7, 20–22]. In addition, the proximal femur in any breed may experience an impressive increase in mineral deposition after malunion fracture or as a consequence of alterations in loading associated with canine hip dysplasia [23, 24]. This increase in mineral deposition is identified radiographically as an increase in proximal femoral medullary bone opacity and is often referred to by the THR surgeon as “proximal femoral sclerosis”. The sclerotic femur presents unique challenges during cementless THR, as the tooth design of the current press-fit THR broaches perform poorly when engaging sclerotic bone. This leads to increased broach strike force, prolonged broaching, excessive use of broaches as files to focally deplete bone, and an increased risk of intraoperative femur fracture [20, 21, 25]. Regrettably, development of canine THR broaches with design features tailored for sclerotic bone has not received attention to date as has been the case for human THR [15]. The current crushing tooth design and broach geometry for the Biomedtrix BFX® press-fit, cementless THR system has remained unchanged since circa 2005 [12].

Given these challenges, a clinical need exists for the development of cementless broaches with design features that facilitate the preparation of sclerotic (i.e. corticalized or excessively dense) femoral bone while simultaneously minimizing the risk of iatrogenic femur fracture. Importantly, the downstream effects of altered broach design on cementless THR stem insertion and subsidence must also be considered. Our long-term goal is to develop femoral broaches that meet these design features for use in the clinical setting. The objectives of this initial proof-of-concept study were two-fold: (1) to evaluate the mechanical performance of three cementless THR broaches in a laboratory setting using simulated normal and sclerotic cancellous bone; (2) to evaluate the effect of broach tooth design on stem insertion and stem subsidence. Bone analogues representing normal and sclerotic canine cancellous bone were prepared for broach insertion using methods identical to those used in the clinical setting during broaching for a Biomedtrix BFX® cementless, press-fit stem. A material testing machine with custom fixtures was used to insert the broaches using a series of constant rate (i.e. quasi-static) sequential drives into the bone analogues. Load-displacement curves were generated. Work-energy integrals were calculated to determine cumulative broaching energy in the simulated normal or sclerotic bone. Finally, the effect of broach tooth design on subsequent cementless stem insertion and subsidence was assessed with the Biomedtrix BFX® press-fit stem using the materials testing machine and custom fixtures fabricated for stem insertion. Refer to the Methods section below for a detailed description of methods. This work represents an important advance in canine cementless THR broach design, which has been static for nearly two decades, and will serve as foundational work for future ex-vivo and clinical studies.

## Results

### Broaching performance

**Fig. 1:**
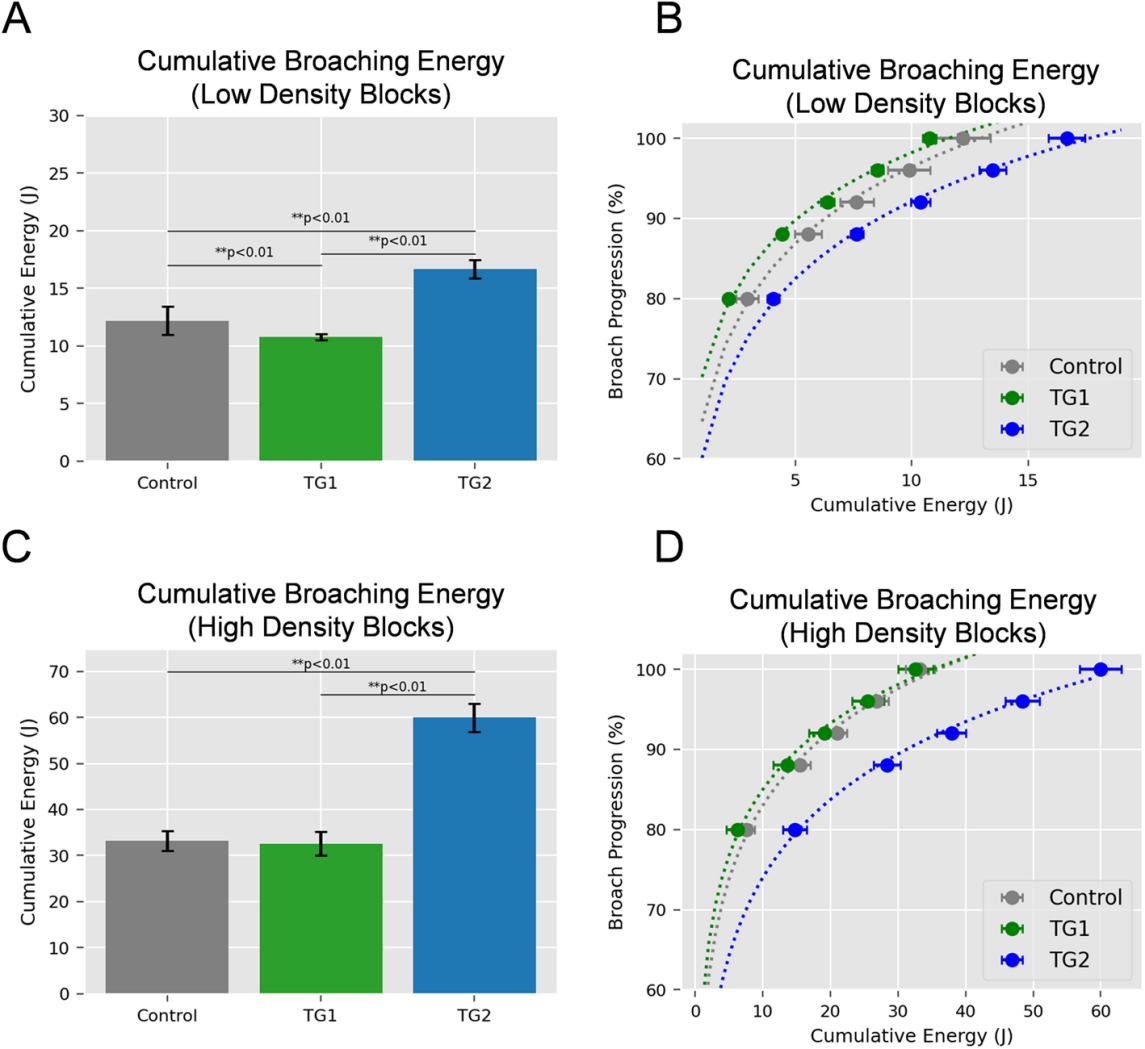
Cumulative broaching energy of three canine cementless THR broach tooth designs in low and high-density test blocks simulating normal and sclerotic bone. Three broach tooth designs were assessed in this study (*n*=3 broaches/group; *n*=3 blocks per broach for both low and high-density test blocks). Low and high-density test blocks were selected to represent normal and sclerotic bone based on the seminal of Goldstein [38]. Broach designs were as follows: Control (in current clinical use), Test Group 1(TG1, additional circumferential V-grooves), Test Group 2 (TG2, diamond tooth pattern) (Figure 3). Low and high density test blocks representing normal and sclerotic bone were prepared for cementless THR broaching using methods identical to cementless THR surgery (described in detail in the Materials and Methods and Figure 4). Blocks were secured in custom fixtures to align with the central axis of the linear actuator and assessed using a series of five sequential drives under quasi-static loading conditions (see Figures 5,6, Table 3). Energy required for broach insertion was determined for low-density blocks simulating normal cancellous bone (**A, B**) or high density blocks representing sclerotic cancellous bone (**C, D**). Cumulative energy across all five drives is reported in A and C. Broaching energy versus percentage broach insertion is reported in B and D. For the low-density blocks, TG1 exhibited the lowest amount of broaching energy. The TG2 broach exhibited the highest broaching energy, regardless of test block density

For the low density test blocks simulating normal cancellous bone, TG1 required the lowest cumulative energy (10.76 ± 0.29 J) during broach insertions, followed by Control (12.18 ± 1.20 J) and TG2 (16.66 ± 0.78 J) (Fig. 1A, B). Significant differences were detected between all groups (*p* < 0.01). For the high-density test blocks simulating sclerotic cancellous bone, TG1 broaches required the lowest cumulative energy (32.60 ± 2.54 J). Control broaches required 33.25 ± 2.16 J and TG2 broaches required 59.97 (± 3.07) J of energy (Fig. 1C, D). A significant difference between the performance of Control and TG1 broaches was not detected (*p* = 0.75). There was a significant difference between TG2 and both Control and TG1 (*p* < 0.01). Peak loads values for each drive are provided in Tables 1 and 2 for low and high density test blocks, respectively. Collectively, these results demonstrate that Control and TG1 broaches required lower energy and lower peak loads for sequential broach insertion regardless of block density, with TG1 broaches exhibiting the lowest insertion energy and peak load values of the three evaluated broach designs. Additionally, the difference in performance between the three broach tooth designs was more evident in the high-density blocks.

**Table 1:** Peak load (kN) values for quasi-static broach insertion (low-density blocks)

	Control		TG1		TG2	
Drive	mean	SD	mean	SD	mean	SD
1	0.40	0.08	0.25	0.05	0.59	0.10
2	0.46	0.04	0.39	0.04	0.69	0.05
3	0.51	0.03	0.46	0.06	0.75	0.08
4	0.55	0.03	0.55	0.07	0.82	0.09
5	0.56	0.02	0.59	0.09	0.89	0.08

Key: Low-density test blocks were prepared as described in Materials and Methods and evaluated using a quasi-static, sequential, five-drive testing protocol. Peak load data for Control, TG1 (V-grooves), and TG2 (diamond pattern) broaches are reported as mean ± SD for each of the 5 drives

**Table 2:** Peak load (kN) values for quasi-static broach insertion (high density blocks)

	Control		TG1		TG2	
Drive	mean	SD	mean	SD	mean	SD
1	0.97	0.13	0.83	0.08	1.76	0.16
2	1.40	0.08	1.37	0.10	2.56	0.28
3	1.60	0.12	1.62	0.21	2.69	0.24
4	1.83	0.09	1.97	0.24	3.02	0.25
5	1.93	0.20	2.24	0.23	3.27	0.29

Key: High-density test blocks were prepared as described in Materials and Methods and evaluated using a quasi-static, sequential, five-drive testing protocol. Peak load data for Control, TG1 (V-grooves), and TG2 (diamond pattern) broaches are reported as mean ± SD for each of the 5 drives

**Table 3:** Quasi-static broach insertion protocol

Drive	Distance traveled (mm)	Net distance (mm)	Percent broach insertion
1	0-60	60	80%
2	0-66	6	88%
3	0-69	3	92%
4	0-72	3	96%
5	0-75	3	100%

Key: Low and high-density test blocks were prepared for cementless THR broach insertion as described in Materials and Methods, Figure 4 and Figure S1. In the clinical setting, a single THR broach is often inserted sequentially in a series of incrementally advancing broach insertion events in which the broach is driven, removed, and re-inserted until the broach is fully seated within the femur. A sequential, five-drive broach insertion protocol was developed to simulate this clinical scenario. Full insertion of the #8 BFX^®^ broach required 75 mm of broach travel. This resulted in complete broach insertion with the shoulder of the broach flush with the chamfer cut of the test block and all broach teeth recessed within the test block (Figure 5D). Specific insertion distances (mm) for each drive are reported above, along with net insertion distance (mm) and percent broach insertion. Note that the travel distance for each drive is not identical. This was intentional in order to simulate the clinical events of femoral broaching, wherein a large travel distance occurs with initial broach insertion and smaller advances are made during the final broach insertion events

### Stem insertion and subsidence performance 

#### Low-density blocks (surrogate for normal cancellous bone)

**Fig. 2:**
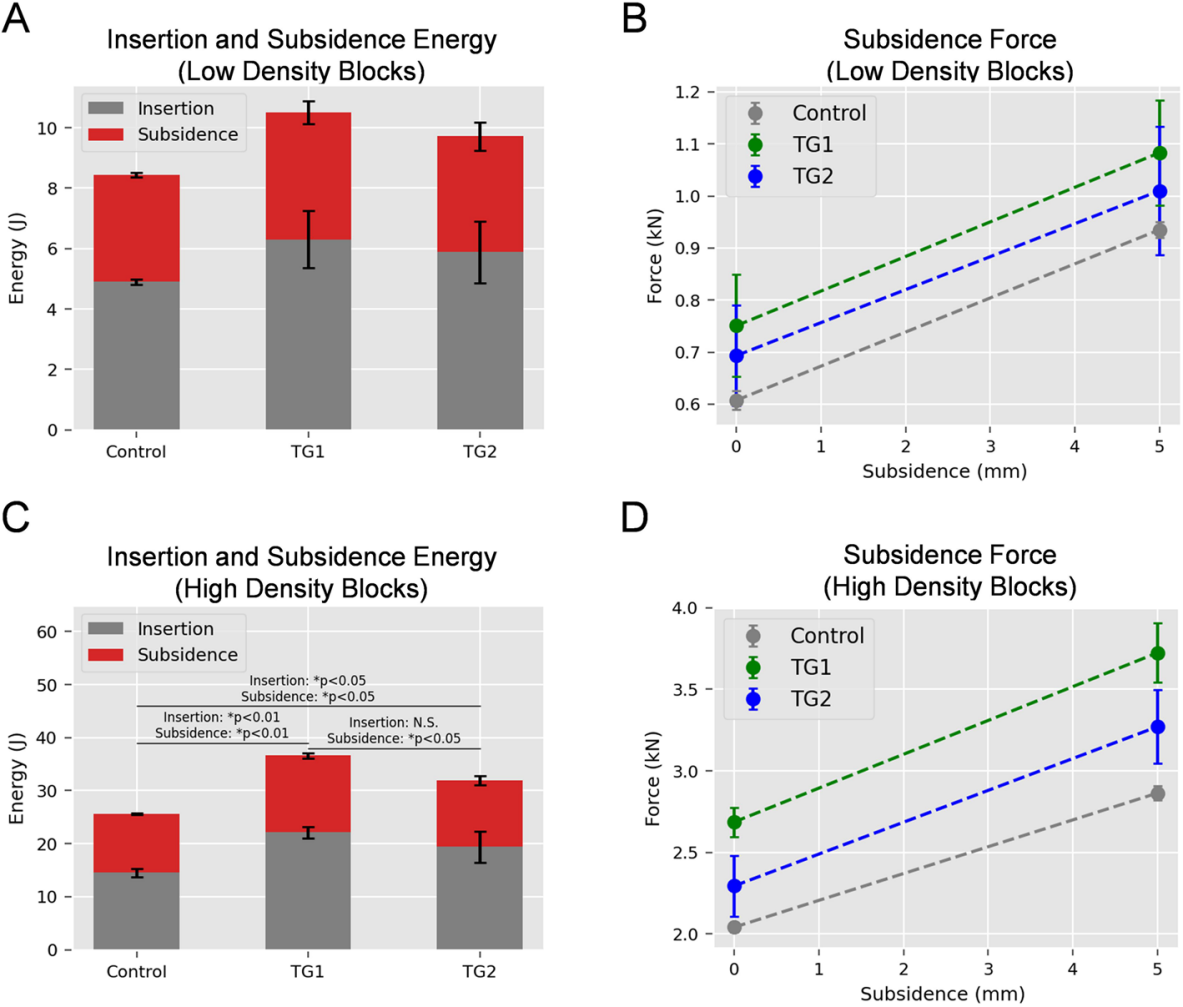
Cementless THR stem insertion and subsidence performance in low and high-density test blocks simulating normal and sclerotic bone. After completion of the broaching protocol reported in Figure 1, low and high-density test blocks representing normal and sclerotic bone were assessed for their resistance to cementless stem insertion and subsidence. A #8 BFX®^®^ stem was secured in custom fixture and aligned with the previously broached test blocks (*n*=3 blocks/condition) as described in Materials and Methods and Figure 7. The cementless stem was inserted at 3 mm/s for a distance of 75mm which represented complete stem insertion with the shoulder of the stem flush with the chamfer cut of the test block. After a five second hold, the stem was inserted an additional 5 mm to represent subsidence (an undesirable event that occurs during the early post-op period after cementless, press-fit THR). Cumulative energy and peak force data were recorded. **A** Stem insertion (gray) and subsidence (red) energy for low density blocks broached with Control, TG1 (V-grooves), and TG2 (diamond pattern) broaches. **B** Subsidence force data for low density blocks from 0 to 5 mm of stem subsidence. **C** Stem insertion (gray) and subsidence (red) energy for high-density blocks broached with Control, TG1, and TG2 broaches. **D** Subsidence force data for high density blocks from 0 to 5 mm of stem subsidence. For both low and high-density test blocks, the TG1 prepared blocks demonstrated the highest stem insertion and subsidence energy and peak force, indicating that blocks of both densities prepared with TG1 broaches provide more resistance during stem insertion and subsidence than blocks prepared with Control or TG2 broaches

While the results presented above suggest that the TG1 broach tooth design may be superior to Control broaches for broaching, the effect of altered broach tooth design on cementless, press-fit stem insertion and subsidence was also assessed. For the low-density test blocks, energy values for stem insertion for Control, TG1, and TG2 were 4.90 (± 0.08) J, 6.3 (± 0.95) J, and 5.88 (± 1.01) J, respectively (Fig. 2A, gray bars). These differences were not statistically significant (*p* > 0.15). Energy values required to achieve 5 mm subsidence for Control, TG1, and TG2 were 3.54 (± 0.07) J, 4.20 (± 0.37) J, and 3.84 (± 0.46) J, respectively (Fig. 2A, red bars). These differences were not statistically significant (*p* > 0.15).

Forces produced at the initiation and completion of subsidence testing are provided in Fig. 2B. While TG1 demonstrated greater force values at the end of stem insertion and initiation of subsidence testing, these values were not significantly different than Control or TG2 (Fig. 2B, 0 mm subsidence, *p* > 0.15). At the completion of subsidence testing, TG1 resulted in higher force values to achieve 5 mm of subsidence, but this value was not significantly different from TG2 or Control broaches (Fig. 2B mm subsidence, *p* > 0.2).

#### High-density blocks (surrogate for sclerotic cancellous bone)

For the high-density test blocks, stem insertion energy for blocks prepared with Control broaches was 14.53 ± 0.81 J, significantly lower than TG1 prepped blocks, 22.53 ± 1.04 J (*p* < 0.01), and TG2 prepped blocks, 19.38 ± 3.00 J (*p* < 0.05). A significant difference was not detected between TG1 and TG2 (*p* = 0.25). (Fig. 2C, gray bars). For subsidence testing, TG1 prepped blocks required 14.49 ± 0.49 J to undergo 5 mm of subsidence, which was significantly greater than test blocks prepared with Control (11.09 ± 0.09 J, *p* < 0.01) or TG2 (12.57 ± 0.81 J, *p* < 0.05) broaches (Fig. 2C, red bars). Blocks prepared with TG2 broaches were also significantly different from Control prepped blocks (*p* < 0.05).

Force data at the initiation and completion of subsidence testing for the high-density test blocks are provided in Fig. 2D. At the end of stem insertion, TG1 prepped blocks exhibited the highest average peak force of 2.69 ± 0.90 kN, significantly more than both Control (2.04 ± 0.31 kN, *p* < 0.01), and TG2 (2.23 ± 1.86 kN, *p* < 0.05) (Fig. 2D, 0 mm subsidence). A significant difference was not detected between Control and TG2 (*p* = 0.09). At the completion of subsidence testing, TG1 prepped blocks exhibited the highest average peak force of 3.72 ± 0.18 kN, significantly more than both Control (2.86 ± 0.04 kN, *p* < 0.01), and TG2 (3.27 ± 0.23 kN, *p* < 0.05) (Fig. 2D mm subsidence). The difference between force at 5 mm subsidence between Control and TG2 was not significant (*p* = 0.058). ​​Collectively, these data suggest that femoral test blocks prepared with TG1 and TG2 broaches required more energy for stem insertion and subsidence. Additionally, these results indicate test blocks prepared with TG1 broaches exhibited the most resistance to stem insertion and subsidence. This was particularly in the high-density test blocks selected to simulate sclerotic femoral bone.

## Discussion

**Fig. 3:**
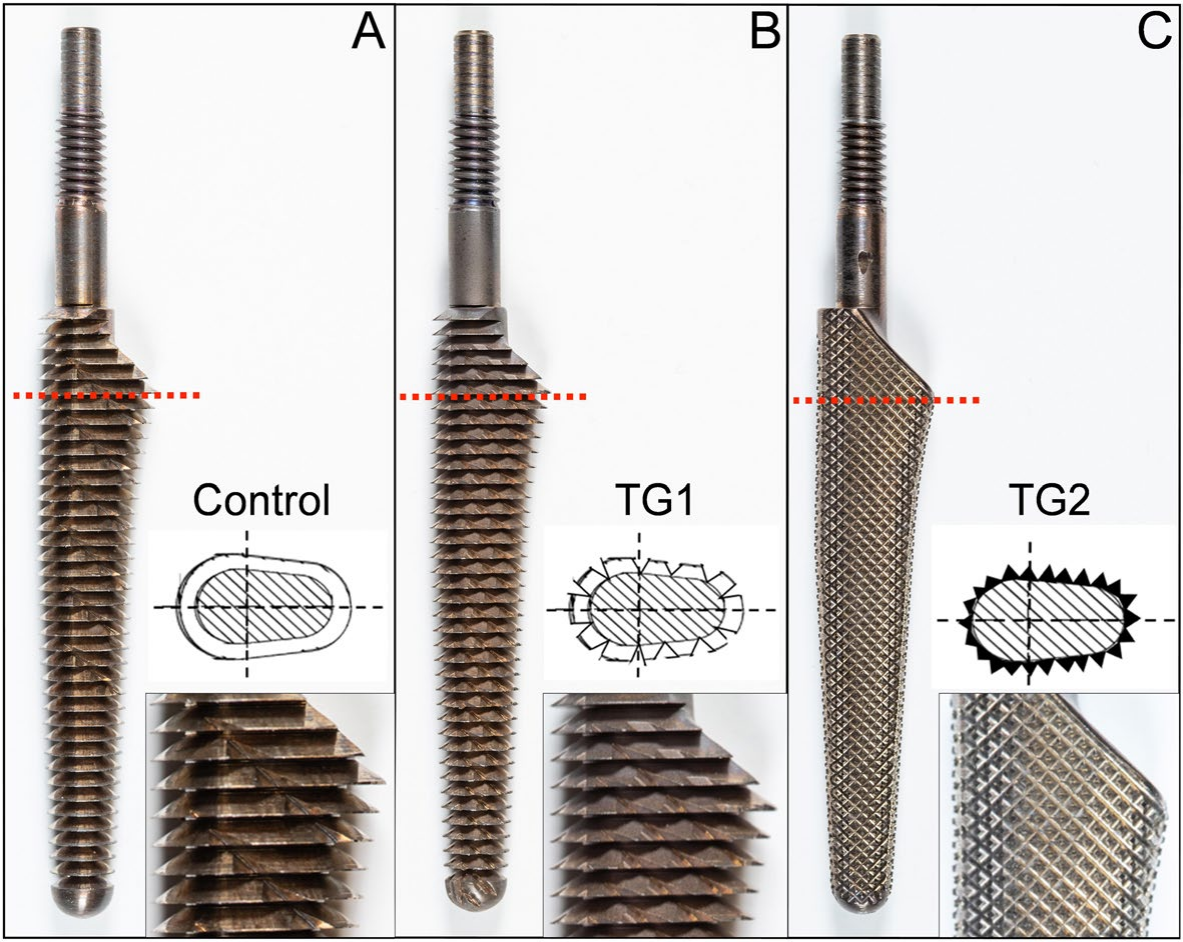
Canine cementless THR broach design. Three canine cementless THR broaches were selected for evaluation. Nine broaches were manufactured in the facility used to generate broaches for clinical use (*n*=3 broaches/group). **A** The control broach (Control) was the standard BFX® design that has been in use since 2005. The broach contains crushing teeth to compact cancellous bone and produce bone densification at the margin of the femoral preparation. **B** Test group 1 (TG1) was designed with identical tooth pitch, chip chamber depth, and rake angle to control broaches, with the addition of 60º V-grooves, 1.5 mm depth, cut parallel to the long axis of the broach circumferentially around the entire broach. These additional V-grooves provide more cutting angles and allow improved clearance of debris. **C** Test group 2 (TG2) was designed with sharp, cutting teeth created in a diamond tooth pattern with a 1.1mm tooth pitch and 0.5 mm chip chamber depth. A diamond tooth pattern provides a sharper cutting tooth than either Control or TG1 broaches while maintaining a precise external broaching envelope. External dimensions of all broaches were identical and all broaches were inspected and conformed to clinical manufacturing tolerances. The shaft of each broach was externally threaded (1/4”-20 UNC) to interface with mechanical testing fixtures

The long-term goal of this research program is to develop improved broaches for canine THR, particularly for challenging cases such those with sclerotic (i.e. corticalized) proximal femurs. The objective of this initial study was to evaluate the mechanical performance of three different canine cementless THR broach tooth designs using quasi-static methods that simulate the clinical process of femoral broaching using test blocks selected to simulate normal or sclerotic cancellous bone. Upon completion of the broach tests, a femoral stem was inserted into test blocks and driven to subsidence in order to determine the effect of alterations in broach tooth design on cementless stem insertion to a desired position (i.e. press fit) or an undesired position (i.e. stem subsidence). TG1 broaches required lower energy for broaching in both normal and sclerotic bone substitutes. TG1 broaches also exhibited peak forces during each of the 5 sequential insertions that were lower or similar to Control broaches in simulated normal and sclerotic test blocks. When stems were inserted into test blocks prepared with TG1 broaches, energy required for stem insertion and subsidence were the highest. The TG1 broach afforded an average 18.7% (low-density blocks) to 30.6% (high-density blocks) greater subsidence resistance when compared to Control broaches. When considered in conjunction with the broaching performance results in Fig. 1; Tables 1 and 2, TG1 broach tooth design exhibited improved broaching performance and also produced press-fit envelopes that were most resistant to stem insertion and subsidence. Thus, based on this initial proof of concept study, the broach tooth modifications of TG1 broaches (Fig. 3B) may represent an important advance in canine cementless THR broach tooth design for this specific THR system. One potential explanation for the improved performance is the cross-sectional area and tooth pattern of the TG1 broaches compared to the cross-sectional area of the BFX® stem. Vertical ridges that corresponded to the V-grooves of TG1 (Fig. 3B) were visually observed in the preparation beds of the test blocks broached with the TG1 broaches. Thus, the lower work energy required for insertion of TG1 is likely explained by the fact that the volume of cut material may be slightly less than that of Control broaches. Secondly, the increased stem stability (higher energy required for stem insertion and subsidence) is likely due to the presence of these ridges during stem insertion. In the clinical setting, it is also possible that the presence of these ridges adjacent to the stem after successful stem insertion will likely lead to enhanced densification adjacent to the stem [15], and a resultant increase in stem stability in the early post-operative period when stem subsidence most commonly occurs [18]. However, this must be assessed in future work and is not supported by the results of this study. The present study describes our initial mechanical findings with simulated cancellous bone substitutes and should not be over-interpreted in regards to clinical performance. Additional work is necessary to determine if these preliminary findings will translate to the clinical setting.

Interestingly, TG2 broaches were designed with a diamond tooth pattern for enhanced cutting of both normal and dense cancellous bone. However, the TG2 broaches exhibited the highest amount of energy for broach insertion for both test block densities. In stem insertion and subsidence testing, TG2 prepared blocks exhibited improved performance when compared to Control test blocks (higher energy required for stem insertion and subsidence), but were inferior to TG1 prepared test blocks. The likely explanation for these results is the low profile cutting teeth on TG2 (Fig. 3C). Tooth chamber depth for TG2 broaches was smaller than Control and TG1 broach designs. It is likely that this design led to rapid teeth filling with bone substitute debris and immediate loss of cutting ability. It is possible that future broach tooth designs using a diamond tooth pattern with a larger chamber depth will address this issue and are likely to be evaluated in the next phase of this research project.

During femoral canal preparation in canine cementless THR with the press-fit system used in the present study, a single set of THR broaches are primarily used as traditional broaches impacted via mallet to sequentially enlarge the femoral preparation bed [12]. However, a secondary use of the same instruments is to use the broaches as files to selectively deplete dense bone in specific regions of the preparation envelope. When resistance to final broach insertion is encountered during canine cementless press-fit THR, the broach is removed and a slightly undersized broach is used to file bone from specific areas of the femoral preparation. This focal depletion of bone then allows the final broach to be re-inserted via impaction to a deeper position within the femur. In some instances, this process is repeated until the final broach is fully seated to the desired position within the femur.

From an engineering standpoint, broaching is a precise, predictable process by which an incrementally enlarging, toothed, tool is driven through a material (in this case bone) to remove material from the preparation site without inducing failure of the material undergoing broaching [26]. Broaching is particularly adapted to the finishing of irregular-shaped envelopes such as encountered in the proximal femur during THR. In contrast, the process of filing, which is similar to sawing or grinding, is a more versatile method used in finishing operations. Filing is used for localized deburring of smaller volumes of material [27]. In the clinical setting of canine cementless THR, the use of a single set of instruments (cementless THR broaches) for both broaching and filing is not ideal; however, it is standard practice due to the cost associated with veterinary THR instrumentation and the need to store, maintain, and service an additional set of THR files. The concepts of broaching versus filing are relevant to the results of the present study, particularly the findings for the TG2 broach tooth design. While TG2 broaches did not perform well in the quasi-static broach testing, this does not indicate that a broach with a similar tooth design to TG2 will not work well if used as a file.

As noted above, additional work is necessary prior to making alterations in broach tooth design in the clinical setting. The next phase of this research program will involve development of an impaction testing system in which THR broaches with varying tooth designs will be impacted in a manner that mimics the method by which THR broaches are clinical inserted (i.e. using a THR mallet and rapid impaction). We considered whether to begin this research program with development of an impaction system. Determining what forces are applied to a broach during clinical mallet-driven impaction during canine cementless THR is not a trivial manner. While it was previously reported that the average impaction force in human femurs was 9.25 kN [28], the forces applied to canine THR broaches are currently unknown and likely vary widely based on dog size, femoral properties, and individual surgeon training and preferences. A study to determine mean impaction force during canine cementless THR is currently in progress. Results of that study will allow more clinically-relevant impaction forces when impaction broach tooth testing is initiated.

### Limitations

As with all studies, the present study is not without limitations. One important limitation preventing direct translation to the clinical setting is the quasi-static testing methodology. Clinically, broaches and stems are inserted by repeated striking with a mallet. Such impact loading is significantly different from the quasi-static methods used in the present study. Non-impact tests, however, are routinely used for initial biomechanical evaluation of broach performance in advance of future clinical applications [29] in part because non-clinical mechanical broaching is nearly universally conducted by non-impact methodologies [30–32]. As the forces applied to canine cementless THR broaches and stems is currently unknown, this initial proof of concept study was performed under quasi-static testing to determine if TG1 and TG2 broach tooth designs warranted further investigation and clinical development. Results of this study clearly demonstrate a difference in both peak loads and cumulative energy for both TG2 and TG2 as compared to Control broaches, with TG1 appearing to have the most promise for future clinical applications. However, due to the differences between clinical broach impaction and quasi-static testing, we were unable to determine whether or not alterations in broach tooth design had an effect on the incidence of fissure.

Another limitation is the selection of polyurethane foam bone substitutes for biomechanical testing. As has been extensively documented in the literature, foam analogues are useful approximations of bone but are significantly different in several ways [33, 34]. For example, bone is a hydrated, viscoelastic, anisotropic material. The proximal femur is a complex geometric structure composed of cortical and cancellous bone and contains an elegant microstructural trabecular design. This complex structure cannot be simulated by polyurethane foam blocks; moreover, the material properties are likely to exacerbate the mechanical difference in impact protocols. However, it is standard practice to use these bone substitutes for exploratory work such as the present study [28, 34–37]. Future studies will focus on development of a synthetic proximal femoral broaching model that simulates the geometry and mechanical properties of the cortex and cancellous bone of both healthy and sclerotic canine femora (allowing assessment of hoop stress in a clinically-relevant manner), determination of impaction loads during clinical broaching and stem insertion, and development of a cementless THR broach impaction testing methodology. The authors caution against translating the findings of this initial proof of concept study directly to the clinical setting.

## Conclusions

In conclusion, the present study describes a quasi-static testing methodology of three canine cementless THR broach design in simulated healthy and sclerotic bone substitutes. Bone substitute test blocks were prepared with methods that closely mimic clinical application of canine cementless THR stems. The test broaches were assessed for peak load and energy during broaching. Lastly, the broached test blocks were assessed for resistance to stem insertion and subsidence. The low-profile diamond tooth pattern of TG2 exhibited inferior performance as compared to Control and TG1 broaches, suggesting that this specific V-groove design may not facilitate broaching of sclerotic bone due to rapid filling of the teeth with debris. The modified V-grooves of TG1 demonstrated superior performance in regards to ease of broaching and resistance to stem insertion and subsidence, suggesting these broach teeth may prove to be an important advance in canine THR. The results of this study will be used to inform broach tooth design in the next phase of this research program, in which an impaction testing system that more closely mimics the clinical impaction of broaches with a THR mallet will be used to determine the biomechanical properties of various broach tooth designs first in bone substitutes and then in cadaveric femurs.

## Methods

### Broach design and fabrication

Broaches were designed and fabricated by Biomedtrix (Whippany, NJ, USA) using manufacturing processes similar to commercially available BFX® broaches. Size 8 broaches were selected for investigation in the present study (*n* = 3 broaches per design) due to the frequency with which these broaches are used in clinical cases. The control broach (Control) was the standard BFX® design that has been in use since 2005 (Fig. 3A). This design contains compaction (i.e. crushing) teeth to compact cancellous bone and produce bone densification at the margin of the femoral preparation [15]. Test group 1 (TG1) was designed with identical tooth pitch, chip chamber depth, and rake angle to control broaches, with the addition of 60º V-grooves, 1.5 mm depth, cut parallel to the long axis of the broach circumferentially around the entire broach (Fig. 3B). The V-grooves provide additional cutting angles and allow improved clearance of debris. Test group 2 (TG2) was designed with sharp, cutting teeth created in a diamond tooth pattern with a 1.1 mm tooth pitch and 0.5 mm chip chamber depth (Fig. 3C). The diamond tooth pattern provides a sharper cutting tooth than either Control or TG1 broaches while generating a precise external broaching envelope. External dimensions of all broaches were identical. All broaches were inspected to confirm clinical manufacturing tolerances were achieved. The shaft of each broach was externally threaded (1/4”-20 UNC) to interface with mechanical testing fixtures (Fig. 3).

### Selection and initial preparation of bone substitutes

Solid rigid polyurethane foam blocks (130 mm x 80 mm x 40 mm) were acquired from a commercial vendor (Sawbones, Vashon Island, Washington, USA). Two foam densities were selected in order to simulate varying density of canine cancellous bone. The 15 pounds per cubic foot (pcf) foam (product #1522-02, 4.9 MPa compression strength, 123 mPa compression modulus) was selected to simulate material properties of normal canine cancellous bone based on previous work [38–40]. The 25 pcf foam (product #1522 − 660) was selected to simulate material properties of dense cancellous bone associated with proximal femoral sclerosis [38]. This 25 pcf foam exhibited a 2.7 fold increase in compressive strength (13 MPa) and a 2.6 fold increase in compression modulus (317 MPa) [41]. The mechanical properties of the 25 pcf foam were consistent with that of dense/sclerotic human cancellous bone but were well below the mechanical properties of cortical bone [38–40, 42].

Stock polyurethane blocks were first modified with a 45° chamfer cut to generate a surface analogous to the femoral neck ostectomy that is created during cementless THR prior to initiation of broaching (Figure S1). Blocks were then sub-sectioned into individual test blocks (130 mm high x 29 mm long x 40 mm wide). A 5 mm diameter channel was drilled through the long axis of each block, centered 12.7 mm from the shoulder of each block. Each channel was sequentially enlarged in 1 mm increments to a final diameter of 8 mm. A Biomedtrix #7 fluted reamer was centered on the 8 mm channel and inserted under power until the reamer was seated with the ¾ reamer line flush with the simulated neck cut, matching the clinical process used to place Biomedtrix cementless stems in clinical cases (Figure S1).

**Fig. 4:**
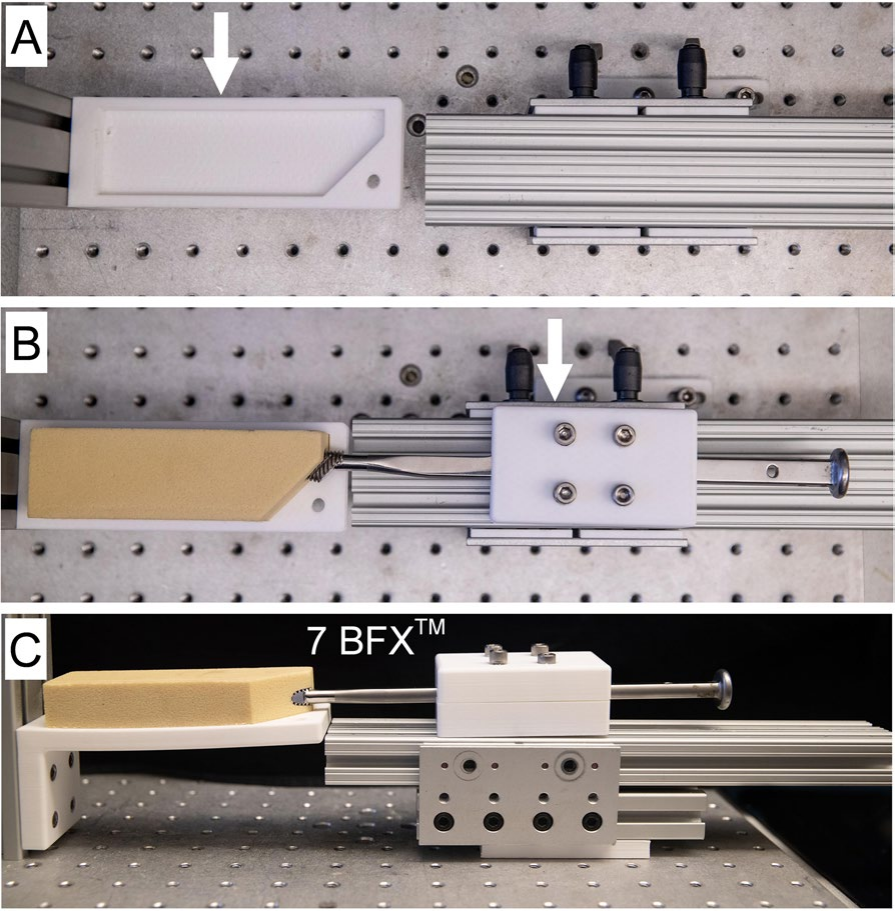
Preparation of low and high-density test blocks prior to the quasi-static, sequential broach insertion protocol. Low and high-density test blocks were prepared using techniques similar to those used in the clinical setting during canine cementless THR. Blocks were cut, drilled to 8 mm, and prepared with a Biomedtrix #7 fluted reamer as descried in Materials and Methods and Figure S1. **A** Next, a custom preparation device was fabricated using aluminum breadboard base and a 3D printed polylactic acid (PLA) test block fixture designed to align and stabilize each test block (arrow) while axially aligning broaches with the center of the canal in each test block. **B** Blocks were secured in the PLA fixture and a size 5-7 BFX^®^ broaches were sequentially secured in a custom-designed 3D printed PLA broach handle fixture (arrow) and fully inserted into each best block with a THR mallet. **C)** The #7BFX® broach was the final broach used to prepare each test block prior to quasi-static testing with the #8 BFX^®^ broaches. A #7BFX^®^ broach is shown fully inserted from a side view

Prior to biomechanical testing, additional preparation was performed on each test block to mimic BFX® workflow for clinical cases. Blocks were placed in a custom preparation device composed of an aluminum breadboard base (#MB1218U, Thorlabs, Inc., Newton, NJ, USA) with a custom-designed, 3D printed polylactic acid (PLA) test block fixture on one end (Fig. 4A). This fixture served to secure each test block and align the long-axis of the previously established 8 mm diameter channel with a linear bearing fixture (#6733, 80/20 Inc., Columbia City, IN, USA) centered on the test block channel. Size 5–7 BFX® broaches were then sequentially secured within a custom-designed 3D printed broach handle fixture and inserted in ascending order using a BFX® THR mallet until the shoulder of the broach was flush with the simulated neck cut. The 7 BFX® broach was the final broach used to complete the test block preparation (Fig. 4B, C).

### Quasi-static evaluation of THR broaches

**Fig. 5:**
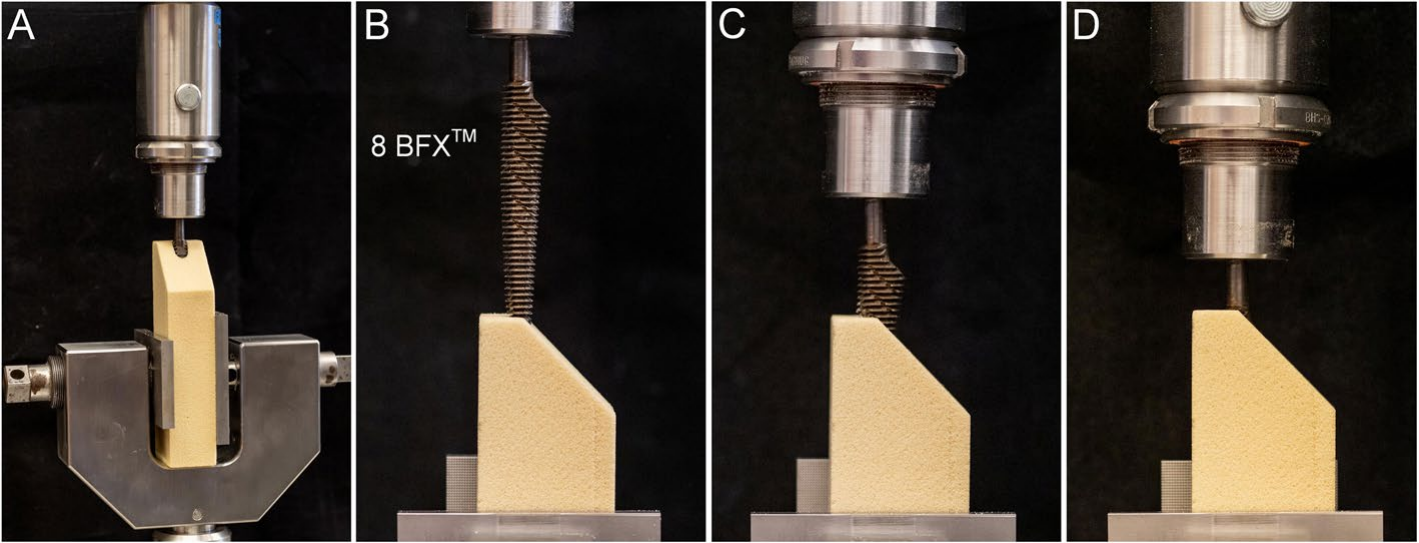
Quasi-static insertion of cementless THR broaches. **A** Custom fixtures were fabricated to secure each test block for mechanical testing. Test fixtures ensured that the center of the actuator and central canal of each test block were aligned. **B** Cementless THR broach shafts of the #8 BFX^®^test broaches were threaded to interface with the testing fixture. Broaches were permitted to rotate freely to self-align during insertion into the test blocks. **C** A cementless broach during quasi-static testing. **D** The cementless broach is shown fully inserted at the completion of the final insertion of the five-drive testing protocol

**Fig. 6:**
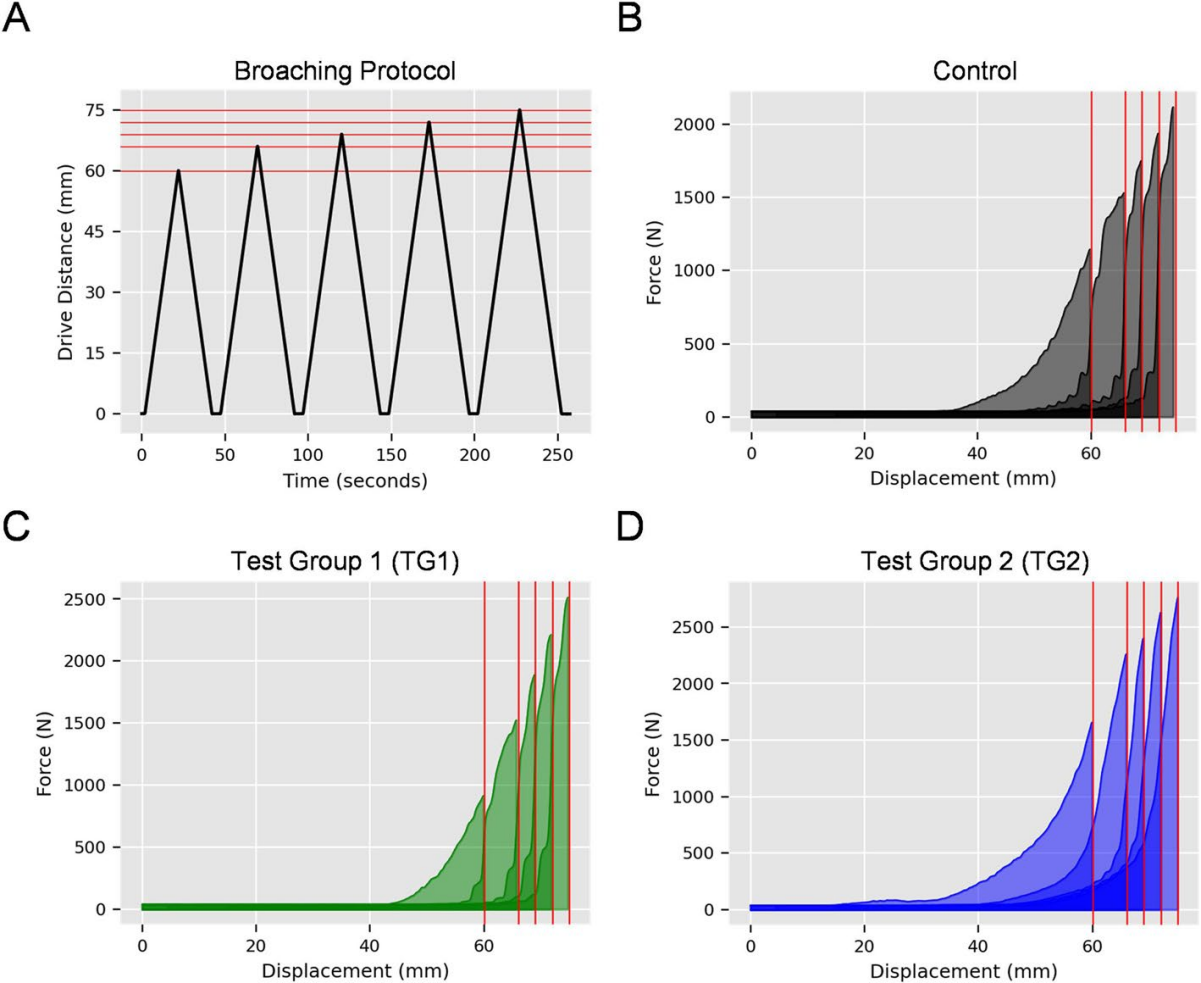
Quasi-static broaching protocol and representative results for the three broach tooth designs. **A **To simulate the manner in which cementless THR broaches are inserted in the clinical setting, a sequential, five-drive loading protocol was developed with each subsequent broach insertion driving the broach farther into each test block. Peak insertion depth is provided for each drive to demonstrate sequential insertion to a final distance of 75 mm which represented complete insertion of the BFX^®^ broach within the test block (shoulder of broach flush with chamfer cut of test block, no broach teeth visible). Exact drive distances are provided in Table 3. **B-D** Representative force vs. displacement graphs for low density blocks broached with Control, TG1, or TG2 broaches, respectively. Energy for each drive is represented by the area under the curve.

Prepared test blocks were secured in a materials testing machine equipped with linear actuator (Thompson Linear, Inc., Radford, VA, USA) for quasi-static broach insertion and removal. Blocks were secured with stainless steel clamps to align each test block preparation bed with the central axis of the actuator (Fig. 5A). Individual test broaches (Control, TG1, TG2; *n* = 3 broaches/group) were secured to the actuator by the threaded end and allowed to rotate freely to self-align during insertion into the test blocks (Fig. 5B, C). Full insertion was defined as 75 mm, corresponding to the length of a complete set of cutting teeth for the 8 BFX® broach (Fig. 5D, Table 3). In order to simulate the clinical scenario of sequential broach insertion, removal, and cleaning, loading was performed in a series of five drives, each followed by extraction at a constant rate of 3 mm/sec. Using displacement control, each drive started at 0 mm then advanced a pre-established distance to mimic the clinical broaching process (Table 3; Fig. 6A). Debris from the test block was cleaned from the broach during each extraction. Load and displacement were measured with a 500 lbf load cell (Futek, Inc., Irvine, CA, USA) and a 15 in. stroke LVDT (TransTek, Inc., Ellington, CT, USA), respectively. The peak load for each drive was reported and energy required to insert the broach was calculated via a work energy integral across the duration of each drive. Cumulative energy was calculated by summing the drive energy across all five drives. Because of the identical external geometry of each broach, it was assumed the same volume of bone material was being removed for each drive, thus the difference in cumulative energy is directly proportional to cutting efficiency. Three test blocks of each density were evaluated for each broach (*n* = 3 broaches per condition) for a total of *n* = 9 test blocks for each broach design. Thus, 27 low-density test blocks and 27 high-density test blocks were evaluated. Representative load-displacement curves for Control, TG1, and TG2 broach configurations are provided in Fig. 6B-D respectively.

### Stem insertion and subsidence

**Fig. 7:**
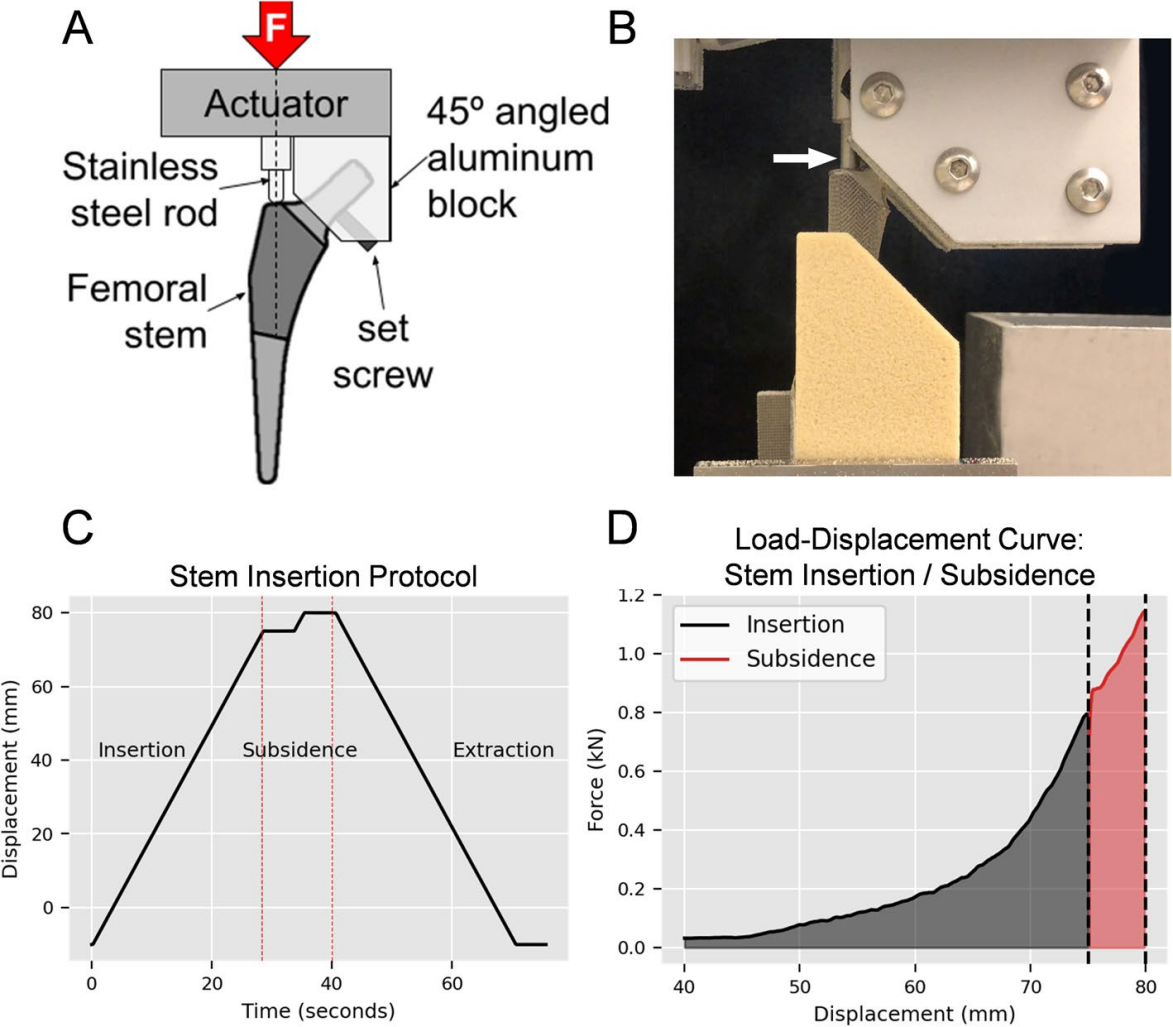
Experimental design for BFX^®^ cementless stem insertion and subsidence testing. **A** A custom fixture was fabricated to accommodate a #8 BFX^®^ cementless THR stem. After securing the stem to a 45° angled aluminum block, a stainless steel rod was positioned in the driver recess of the cementless THR stem. Stem alignment with the previously broached test block was verified with a digital protractor and the stem was fixed in place with a set screw. As with broach testing, free rotation about the loading axis was permitted to allow the stem to self-align with the preparation bed of each test block. **B** Representative photograph of the #8 BFX^®^ stem secured within the custom fixture during insertion into a test block. Note that the stem is loaded by the stainless steel rod (white arrow) at the driver recess of the stem in a manner identical to stem insertion in clinical cases. **C** Stem insertion and subsidence testing protocol, demonstrating a 75 mm insertion test, a five second pause, an additional 5 mm subsidence test, followed by a pause and stem extraction. **D** Representative force vs. displacement graph of the BFX^®^ stem during insertion and subsidence. Energy is represented by the area under the curve for both insertion (gray) or subsidence (red). Note the increased energy present during the relatively small 5 mm subsidence test distance

To assess the effect of the three broach tooth designs on cementless stem insertion and subsidence, a #8 electron beam melted (EBM)-BFX® femoral stem (Biomedtrix, Inc.) was inserted into broached test blocks using a custom fixture connected to the linear actuator (Fig. 7A, B). This fixture consisted of a round tip stainless steel rod that was positioned coaxial with the linear actuator to interface with the driver recess of the femoral stem. This ensured the applied compressive force was coaxial with the stem. Two 45° flat plates (#4145, 8020 Inc.) and aluminum extrusion components were combined to create a 45° angled aluminum block that was secured to the neck of the stem. Once in place, the stem alignment with the actuator axis was verified with digital protractor, then fixed in place with a set screw (Fig. 7A). Free rotation about the loading axis was permitted for the entire apparatus to allow the stem to self-align with the preparation bed of each test block in a manner identical to broach insertion.

Stem insertion proceeded at a rate of 3 mm/s for a distance of 75 mm such that the proximal aspect of the stem was flush with the simulated neck cut. After a five second hold, the stem was driven an additional 5 mm at 3 mm/s to simulate stem subsidence in the early post-operative period (Fig. 7C, D). Upon the completion of each stem insertion test, the BFX stem was extracted from the test block at 3 mm/s then cleaned with a nylon brush and compressed air to remove accumulated foam block debris. To minimize the effect of stem re-use, a representative sample (*n* = 3) of the broached test blocks were used for each broach design for both test block densities. The order in which test blocks were evaluated was randomized. Peak force and cumulative energy were calculated for both insertion and subsidence and used as a proxy for stem stability (i.e. resistance to subsidence).

### Statistical analysis

All datasets were examined for normality using the Kolmogorov-Smirnov test of normality. Normally distributed data were reported as mean and standard deviation. Peak force in kilonewtons (kN) and cumulative energy (J) were analyzed using one-way ANOVA with Tukey HSD post hoc analysis for broaching, stem insertion, and subsidence data. Python 3.7 (Python Software Foundation, Beaverton, OR, USA) was used for descriptive and analytical statistics. Significance was assumed at *p* ≤ 0.05.

### Supplementary Information

Supplementary Material 1.

## Data Availability

The datasets generated and/or analyzed during the current study are available from the corresponding author on reasonable request.

## Abbreviations

BFX^®^: Biologic fixation, or system-specific nomenclature for cementless total hip replacement instrumentation and implants
CFI: Canal flare index
J: Joule(s)
kN: Kilonewton
pcf: Per cubic foot
PLA: Polylactic acid
TG1: Test group 1
TG2: Test group 2
THR: Total hip replacement
