# Correction: Quasi-static mechanical evaluation of canine cementless total hip replacement broaches: effect of tooth design on broach and stem insertion

**DOI:** 10.1186/s12917-024-04122-8

**Published:** 2024-06-19

**Authors:** Zachary T. Lawson, Danielle L. Hollenbeck, Catrina J. Silveira, Michael R. Moreno, Andrew B. Robbins, W. Brian Saunders

**Affiliations:** 1 College of Engineering, Texas A&M University, College Station, Austin, TX USA; 2 Department of Small Animal Clinical Sciences, College of Veterinary Medicine and Biomedical Sciences, 4474 TAMU, College Station, TX 77843‑4474 USA

**Correction:** ***BMC Vet Res*** **20, 222 (2024)**

10.1186/s12917-024-04075-y

Following the publication of the original article [1], the authors want to change the “Austin” city in affiliations 1 and 2 with “College Station” city. The original article has been corrected.

Affiliations 1 and 2 in the published version currently reads:

1 College of Engineering, Texas A&M University, College Station, Austin, TX, USA

2 Department of Small Animal Clinical Sciences, College of Veterinary Medicine & Biomedical Sciences, Texas A&M University, 4474 TAMU, College Station, Austin, TX 77,843 4474, USA

Affiliations 1 and 2 in the published version should read:

1 College of Engineering, Texas A&M University, College Station, TX, USA

2 Department of Small Animal Clinical Sciences, College of Veterinary Medicine and Biomedical Sciences, 4474 TAMU, College Station, TX, 77,843 − 4474, USA
