# Supplementary figures and images for: Quasi-static mechanical evaluation of canine cementless total hip replacement broaches: effect of tooth design on broach and stem insertion
Source: BMC Vet Res. 2024 May 24;20:222. doi: 10.1186/s12917-024-04075-y (PMC11118540; doi:10.1186/s12917-024-04075-y)

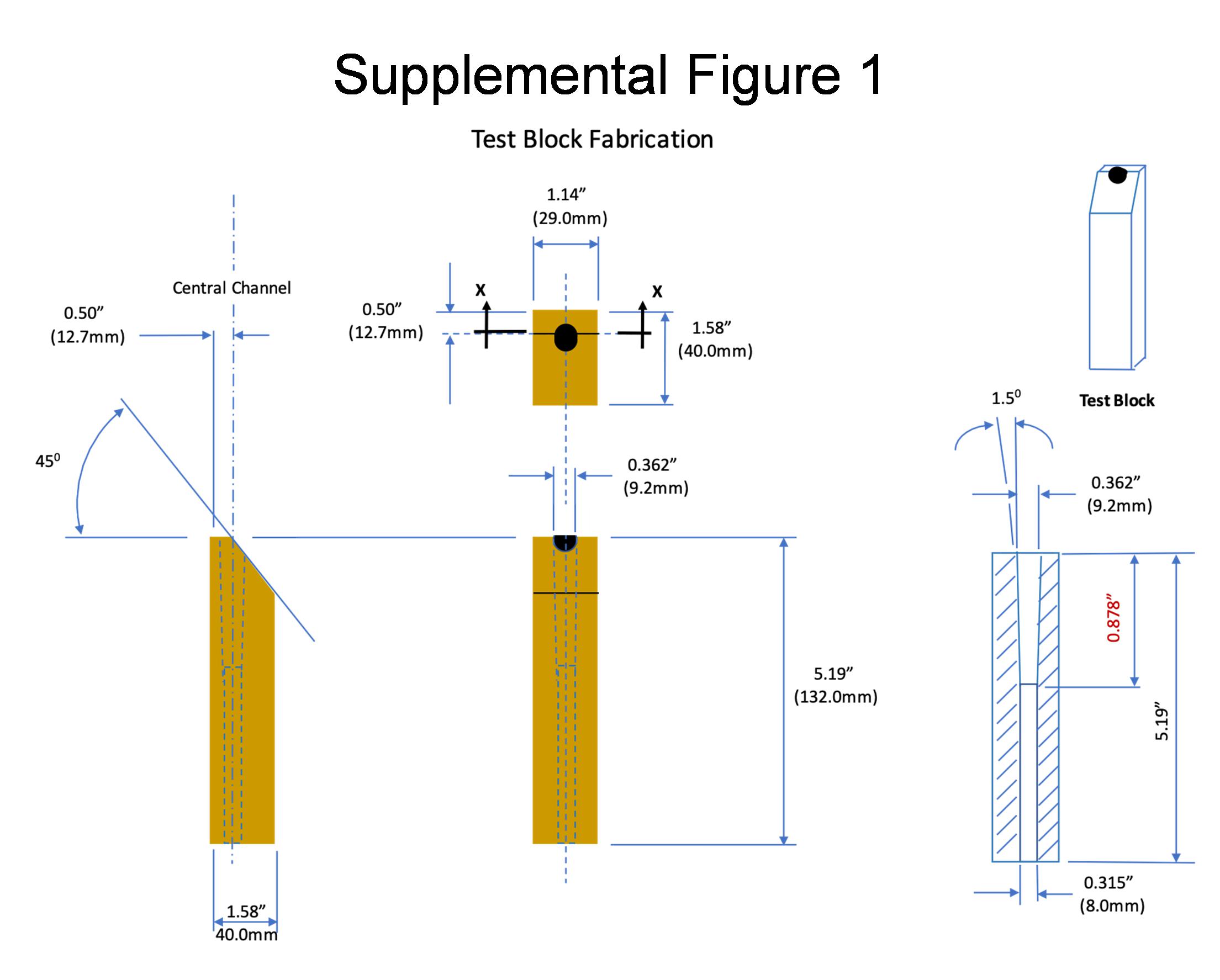

Supplement: Supplementary file 1 — Supplementary Material 1. [file 12917_2024_4075_MOESM1_ESM.jpg]
